# Empathic AI for Patient-Centered Cancer Care: A Scoping Review of Patient Navigation, Support, and Clinical Practice

**DOI:** 10.2196/82336

**Published:** 2026-04-09

**Authors:** Brianna M White, Gabriela L Aitken, Janet A Zink, Parnian Kheirkhah Rahimabad, Fekede Asefa Kumsa, Soheil Hashtarkhani, Rezaur Rashid, Saba Kheirinejad, Tyra Girdwood, Christopher L Brett, Robert L Davis, David L Schwartz, Arash Shaban-Nejad

**Affiliations:** 1 Department of Pediatrics, College of Medicine, The University of Tennessee Health Science Center—Oak Ridge National Laboratory Center for Biomedical Informatics, 50 N Dunlap, 492R, Memphis, TN, 38103, United States, 1 901 287 5836; 2 Departments of Radiation Oncology & Preventive Medicine, The University of Tennessee Health Science Center, Memphis, TN, United States; 3 Department of Community and Population Health, College of Nursing, The University of Tennessee Health Science Center, Memphis, TN, United States; 4 Department of Radiation Oncology, University of Tennessee Graduate School of Medicine, Knoxville, TN, United States

**Keywords:** empathic AI, oncology, cancer care, patient-centered care, AI in health care, digital health, health equity, artificial intelligence

## Abstract

**Background:**

Artificial intelligence (AI) is rapidly reshaping oncology, offering advancements in clinical care and patient support. A growing area of interest is the integration of empathic AI: systems integrating clinical precision with emotional intelligence to support medical decision-making and the emotional and psychosocial well-being of patients and caregivers.

**Objective:**

This review aimed to explore the role of empathic AI in cancer care, with a focus on its applications in patient education, clinician support, and emotional care. It also evaluated the ethical, cultural, and implementation challenges associated with its integration into clinical practice in oncology.

**Methods:**

A systematic search of literature was conducted in accordance with PRISMA (Preferred Reporting Items for Systematic Reviews and Meta-Analyses) 2020 guidelines. Peer-reviewed papers published between January 2018 and January 2025 were identified through a search of PubMed, Scopus, and IEEE Xplore, and citation tracking. Eligible studies focused on applications of empathic AI in oncology. A total of 44 studies were included and analyzed thematically across 3 core clinical domains: tailored communication and education, diagnostics and care plan optimization, and emotional and psychosocial support.

**Results:**

Empathic AI demonstrates the potential to improve cancer care by enhancing patient education, clinical decision-making, and emotional support. Common applications include personalized education for patients and providers, support for diagnostic and therapeutic decisions, and tools designed to recognize and respond to patient distress. Several studies noted improved patient engagement and reduced clinician burden. However, concerns were identified regarding overreliance on AI systems, cultural insensitivity, and patient privacy.

**Conclusions:**

Empathic AI represents a promising advancement in patient-centered oncology, integrating emotional intelligence into clinical care. Its successful implementation will require careful attention to ethical, cultural, and clinical considerations to ensure health equity, trust, and safety in AI-assisted cancer care.

## Introduction

Artificial intelligence (AI) has become increasingly embedded in medical practice over the past decade, enhancing care capabilities from the development of personalized health libraries and innovative patient education strategies to disease screening, diagnostics, and treatment planning [1-4]. These technologies are reshaping how care is accessed, delivered, and experienced. In oncology, AI models are being developed to standardize and improve the consistency and quality of care, offering new ways to support both clinicians and patients in navigating complex diagnostic and treatment pathways [5]. These technologies provide timely, accurate, and accessible information that can empower patients and caregivers, supporting engagement, comprehension, and autonomy throughout the cancer care continuum [6-8].

For example, AI has rapidly become a critical adjunct for medical image analytics, demonstrating that computer models can achieve state-of-the-art tumor detection, segmentation, extraction, and classification across a wide range of cancer types [9]. These advancements in AI in health care have improved diagnostic accuracy and enabled earlier cancer detection, allowing clinicians to initiate timely, evidence-based interventions. Beyond imaging, AI supports personalized oncology through predictive models that generate tailored treatment recommendations based on individual patient demographics, genomics, and clinical profiles [10]. These technologies have ushered in a new era of precision health and medicine [11], specifically impacting the field of oncology, informing care strategies that range from recurrence prediction and surgical planning to emotional assessment. By integrating psychosocial considerations into clinical decision-making, AI holds promise not only for improving treatment efficacy but also for enhancing overall quality of life [10]. This progress may help address systemic gaps in access to emotional support and culturally responsive communication, which have been long-standing public health challenges in cancer care.

However, despite these advances, the integration of AI into emotionally sensitive domains such as oncology introduces a new set of unresolved ethical, relational, and epistemological challenges [12]. The emotional toll of a cancer diagnosis underscores the critical need for empathetic care. Empathy, long considered a distinctly human element of care, has historically been difficult to replicate through technology. Yet recent advancements suggest that AI systems may approximate emotional attunement, recognize distress, and respond in ways perceived as empathic and compassionate [13]. In this context, empathic AI refers to technologies designed to detect and respond to users’ emotional states to foster interactions that feel emotionally responsive and humanlike [13].

This review examines the role of empathic AI in cancer care, with a focus on its potential to enhance patient-centered, holistic treatment. We synthesize current advancements, challenges, and ethical considerations to inform the development of empathic AI systems that support both clinical effectiveness and emotionally responsive care. Given the distinct emotional, ethical, and communicative demands of oncology, this review underscores the need for a field-specific framework to guide the safe, equitable, and meaningful integration of empathic AI into cancer care delivery. From a public health perspective, thoughtful integration can inform scalable interventions, guide equitable policy design, and improve outcomes for historically underserved populations.

## Methods

### Search Strategy

The initial review of literature incorporated peer-reviewed studies that were identified on January 6, 2025, from PubMed, published between January 2018 and January 2025. This timeline was adopted to ensure that the review focuses on the most recent advancements and innovations in the use of empathic AI in cancer care as technological developments are rapidly evolving. Our search terms were used based on the thesaurus and keywords including AI, empathic, empathy, emotion, emotional intelligence, cancer, tumor, and oncology. Terms and keywords were combined to comprise search phrases, allowing for a more sensitive search. Search phrases included “artificial intelligence” OR “AI” AND “empathic” OR “empathy” OR “emotion” OR “emotional intelligence” AND “cancer” OR “oncology”.

Following peer review, the search strategy was expanded to strengthen database scope. An updated search was conducted in Scopus and IEEE Xplore on January 1, 2026, using the same search terms and inclusion criteria, and newly identified studies were screened and extracted using the same procedures as the original search. Studies identified through the updated search were fully integrated into the descriptive and thematic analyses, and totals reflect the combined results of both search phases.

### Eligibility Criteria

Studies were included for review if they met the following eligibility criteria: (1) published from January 2018 to January 2025, (2) explored the relationship between empathic AI and cancer care, and (3) available in the English language. Studies were excluded if they were not original research or were not peer-reviewed.

### Study Selection, Data Extraction, and Analysis

The initial electronic database search yielded 78 results, which were collectively screened by 2 reviewers (GA and JZ) using the eligibility criteria. Following the abstract and full-text screening, 64 studies were eliminated because they did not meet the inclusion criteria. To expand the scope of relevant literature, a snowballing technique was used, where key studies identified during the initial search were used to trace additional sources through references and citations. This process led to the identification of 15 additional studies, enhancing the comprehensiveness of the review. A third reviewer (BW) verified the full set of included and excluded studies, cross-referencing decisions with the original criteria and extraction tables. Any uncertainties or inconsistencies were discussed collaboratively among the 3 reviewers to ensure a consistent and transparent review process. Study quality indicators (ie, peer-review status, study design, and sample size) were collected during data extraction to contextualize findings and mitigate potential sources of bias.

### Terminology

Throughout this review, we use the term “empathic AI” as an umbrella descriptor for AI systems designed to recognize, respond to, or adapt to users’ emotional states in health care contexts. Closely related terms such as empathetic AI or emotionally intelligent AI appear sparingly and only when reflecting terminology used in the source literature. These terms are treated as conceptually overlapping within this review, and their use does not imply substantive distinctions in system function or intent.

## Results

### Overview

**Figure 1.**
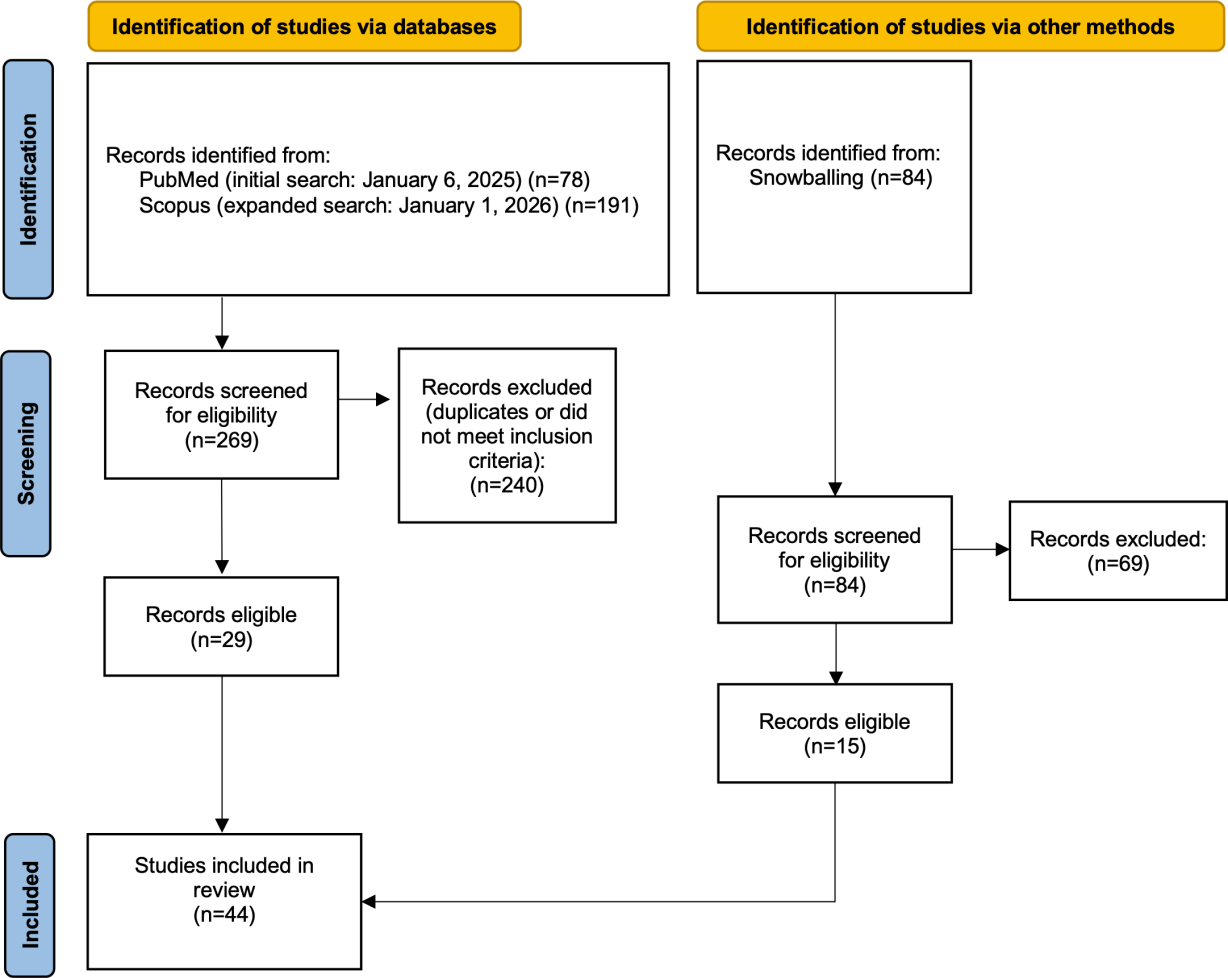
PRISMA (Preferred Reporting Items for Systematic Reviews and Meta-Analyses) 2020 flow diagram of study selection process.

As shown in Figure 1, a total of 44 studies met the inclusion criteria for review. Study characteristics are summarized in Table S1 in Multimedia Appendix 1 [14-57]. The included literature spans from June 2018 to January 2025, reflecting the evolution of empathic AI applications in cancer care over time. A synthesis of key applications, thematic domains, and representative findings across the included studies is shown in Table 1.

**Table 1.** Summary of key findings by clinical domain of empathic artificial intelligence in cancer care.

Domain	Key applications	Representative findings	Included references
Tailored communication and education	Chatbots delivering emotionally calibrated, context-aware patient education LLMs^a^ and NLP^b^ tools supporting empathic provider communication Real-time summary tools and simulation-based training	Improved patient comprehension, reduced distress, and greater engagement in care Enhanced clinician ability to convey complex or difficult information empathetically Reduced provider burnout and communication fatigue	[14-51]
Enhanced diagnostics and treatment planning	Integration of clinical and emotional data for diagnostics and care planning AI^c^-assisted screening, documentation, and decision-making Tailored treatment strategies based on emotional state, literacy, preferences, and trust	Increased diagnostic accuracy and efficiency More personalized and emotionally attuned treatment plans Improved adherence, patient satisfaction, and provider-patient trust	[17,18,20,22,23,25-27,31,34,37-43,45-47,50-54]
Emotional and psychosocial support	NLP and affective computing to detect distress and offer emotional responses Chatbots and support tools applying positive psychology principles LLMs and cofacilitators offering caregiver guidance VR^d^ interventions and NLP tools for end-of-life discussions	Reduced anxiety, isolation, and emotional distress in patients Enhanced emotional support and resilience for caregivers Improved mood, stress reduction, and support in palliative and end-of-life contexts	[14-17,19-36,38,39,41-51,53-56]

a LLMs: large language models.

b NLP: natural language processing.

c AI: artificial intelligence.

d VR: virtual reality.

Thematic analysis was conducted through a qualitative synthesis of the studies shown in Table S1 in Multimedia Appendix 1. Each paper was reviewed to extract key concepts, use cases, and reported outcomes relating to the application of empathic AI in cancer care. To organize these findings meaningfully, we used an inductive approach, identifying patterns that emerged directly from review of the data. These patterns were then organized into three overarching clinical domains: (1) tailored communication and education, (2) enhanced diagnostics and treatment planning, and (3) emotional and psychosocial support. The domains were refined iteratively as themes reoccurred across studies, allowing grouping of findings into categories that reflect the diverse roles of empathic AI in cancer care.

### Tailored Communication and Education

Tailored communication and educational interventions emerged as a central theme across the majority of included studies, underscoring the potential of empathic AI to deliver information that is both clinically relevant and dynamically attuned to the emotional and cognitive needs of both patients and providers.

### Tailored Patient Education

Empathic AI systems supporting personalized patient education were reported in 86% (38/44) of studies, highlighting growing adoption of these technologies to enhance informational access and emotional resonance in patient care. These tools tailor educational content to individual patient contexts, including diagnosis, treatment phase, emotional state, and cognitive load. Chatbots and AI cofacilitators were most often used to simplify complex medical concepts, offer condition-specific coping strategies, and reduce distress through emotionally calibrated messaging.

For example, Greer’s *Vivibot* chatbot leveraged a positive psychology framework to support emotional regulation in young adult cancer survivors, resulting in reduced anxiety and high user satisfaction [14]. Studies by Leung et al [15,16] evaluated AI cofacilitators that adapted group-based educational content in real time based on participant mood and interaction dynamics, enhancing cohesion and engagement. Similarly, Chaix et al [17] and Siglen et al [18] reported that chatbot-delivered education improved adherence to treatment regimens and facilitated greater engagement in shared decision-making. By modifying tone, language, and complexity in real time, AI tools improved patient comprehension and engagement.

Chen et al [19] evaluated the use of AI chatbots such as GPT-3.5, GPT-4, and Claude AI to generate high-quality, empathetic, and readable responses to cancer-related questions posed by patients on social media. The study found that AI-generated responses, particularly those from Claude AI, outperformed those from oncologists in terms of empathy, clarity, and emotional resonance, suggesting that AI chatbots can serve as effective tools for enhancing patient education and communication. Other studies specifically highlighted the role of empathic AI in expanding access to reliable, emotionally attuned health information among historically underserved or low-literacy populations [20,21]. These findings position AI as a tool to enhance patient education equity, particularly in contexts where human resources are limited, or in settings where patients may be hesitant to ask questions directly.

Recent studies further extend this work by demonstrating how generative AI tools tailor educational content based on patient demographics, clinical context, and informational needs, including evaluations of ChatGPT and other large language models (LLMs) responding to cancer-related questions and decision-making scenarios [22-25].

However, concerns about misinformation, cultural insensitivity, and overreliance on AI-generated outputs were raised across multiple studies [26-28]. These findings emphasize the importance of clinical oversight and algorithmic guardrails. Scalability and safety remain central to the integration of AI-mediated education in oncology as these tools move closer to frontline deployment.

### Tailored Provider Education

Empathic AI was also used to support tailored provider education in 52% (23/44) of studies, reflecting a growing interest in leveraging AI to enhance clinical decision-making and support emotionally intelligent communication. These tools were positioned as cognitive extenders, offering context-specific recommendations on treatment protocols, synthesizing complex medical data, and facilitating more empathic responses during patient interactions. As 1 study highlighted, this may be particularly important in high-stress oncology settings for reducing clinician burnout and compassion fatigue [29].

Several studies examined the use of LLMs to help clinicians better understand caregiver perspectives, enabling the cocreation of more responsive group interventions [30,31]. Providers received AI-generated conversation summaries or context-sensitive prompts, which improved their ability to convey difficult information with empathy and precision. Others found that AI-generated patient-facing responses were rated significantly higher in quality, clarity, and empathy than those written by clinicians under time pressure, pointing to the potential of AI to support consistency in emotionally attuned communication [21,28,32]. One study comparing physician and AI chatbot responses with patient inquiries on social media found that AI replies were preferred 78.6% of the time [32].

Furthermore, De Silva et al [33] explored the use of machine learning and natural language processing (NLP) to analyze patient interactions in online cancer support groups. By identifying patient-reported behaviors, emotions, and treatment decisions, AI tools provided valuable insights into patient decision-making, emotional dynamics, and care preferences. The findings highlighted how AI-driven insights could help clinicians better understand behaviors, decisions, side effects, and emotional cues within support groups to derive actionable insights for patient-centered care. Recent work has also explored hybrid and simulation-based approaches, including live-chat models emphasizing digital clinical empathy [34] and virtual patient systems designed to train clinicians in empathic and end-of-life communication [35,36]. Other studies explored the use of empathic AI for real-time feedback and simulation-based training, with AI tools positioned not just as repositories of information but as companions in ethical and emotional reasoning [26,27].

These findings underscore the potential of AI to cultivate emotionally intelligent clinician communication. Nevertheless, integration challenges remain. Several studies raised concerns about how increasing reliance on AI may shift the balance between algorithmic input and clinical judgment, prompting debate about the potential erosion or recalibration of clinical intuition. While some view this shift as a loss, others argue that AI could enhance intuition by providing real-time feedback and mitigating cognitive biases [21,37]. Although AI may reduce cognitive load and offer scalable training solutions, its ability to cultivate true empathic capacity remains contested, which raises important questions for future research and professional development strategies.

### Enhanced Diagnostics and Treatment Planning

The decision-making use of empathic AI in cancer care was explored across several areas, from diagnostics to care plan optimization. In total, 70% (31/44) of the included studies addressed the use of empathic AI in the clinical space, with approximately half examining both diagnostics and treatment planning. Studies in this theme revealed how AI systems are increasingly integrated into clinical decision-making, optimizing care goals and improving patient outcomes.

### Diagnostics and Decision-Making Support

Empathic AI systems have been shown to support clinicians in making more accurate diagnoses and treatment decisions. Among the studies reviewed, 43% (19/44) discussed how AI is being used to aid in diagnostic reasoning and clinical interpretation. By integrating emotional intelligence with clinical data, these tools help enhance the accuracy of patient assessments, considering both clinical factors and the emotional context of the patient’s situation. For example, ChatGPT has been used to assist in the interpretation of personalized breast cancer screening studies with 80% accuracy while reducing clinician workload by 70% [38]. Similarly, these algorithms have supported individualized radiation therapy dosage planning [39], enhancing diagnostic efficiency and precision in a patient-centered manner. While these applications are technically oriented, the authors emphasize that “using AI and combining it with human wisdom, empathy, and affection will be the method of choice for further fruitful development of tomorrow’s senology” [38], highlighting the envisioned role of empathy even within data-driven clinical tools [40,52].

Furthermore, NLP models have been used to analyze patients’ decision-making behaviors and emotions [33,41], allowing clinicians to understand patients better and tailor their recommendations accordingly. When it comes to clinical documentation, AI-powered notes written by OpenAI’s ChatGPT 3.5 and GPT 4.0 have been found to be more personalized and empathetic [42]. Together, these functions of AI platforms create not only a precise and tailored patient experience but also one that is more patient-specific. While AI integration into the clinical realm is in its infancy, it has a definite role in enhancing patient care delivery and optimization.

### Care Plan Optimization

Several studies examined how AI-driven systems can optimize care plans by considering both medical and psychosocial factors. In total, 61% (27/44) of studies addressed how AI enhances patient-centered decisions and care. Specifically, these AI tools assist in tailoring treatment regimens that account for individual patient preferences and emotional well-being, ensuring a more holistic approach to cancer care. For example, AI models such as the Vik chatbot, designed to support patients with breast cancer[17], can personalize conversations and treatment recommendations based on a patient’s literacy level and personal context. This helps patients make informed decisions and improves treatment adherence [57]. Others emphasize the influence of AI-generated guidance on treatment decision-making in advanced or complex cancer contexts, including variability in tone, content, and recommendations that may shape patient choices [22,52]. Furthermore, multiple studies examining chatbots showed that patients are capable of trusting machines [27], including the Rosa chatbot focused on breast cancer gene testing embedded into an app for patients with breast and ovarian cancer [18]. AI can, therefore, be used to detect distress and other emotions as shown by the results of using an AI-based therapy group cofacilitator [16], which allows physicians to tailor their patient-to-provider interactions accordingly, resulting in more patient-centered care. Through these functions, AI platforms foster stronger patient-provider relationships in the clinical landscape [43].

Studies included in this theme demonstrate that AI enhances patient-provider communication and real-time emotional support, leading to optimized care plans. Although concerns regarding machine privacy remain, these studies support the use of AI systems as copilots or cofacilitators in clinical decision-making to enhance the holistic work of physicians.

### Emotional and Psychosocial Support

Another prominent theme in the studies was the emotional and psychosocial support that empathic AI can offer to both patients and caregivers. To underscore the importance and timeliness of this theme, the vast majority of studies (40/44, 91%) alluded to applications of the technology involving patient or caregiver emotional support. These AI systems are increasingly seen as tools for alleviating stress, providing reassurance, and offering emotional guidance during challenging cancer journeys.

### Patient Emotional Support

Several studies focused on the ways empathic AI can provide emotional comfort to patients, offering them a sense of companionship and reducing feelings of isolation. A large majority of studies reviewed (40/44, 91%) highlighted the use of empathic AI in the service of patient emotional support. By using NLP and affective computing, AI systems were able to recognize and respond to emotional cues, providing timely encouragement or referrals to professional counseling services [53,54]. Likewise, Mårell-Olsson et al [44] demonstrated that socially intelligent agents can address nonmedical needs in pediatric populations by reducing feelings of isolation, supporting coping, and facilitating participation in daily activities.

The evaluation of the ability of various recently released AI chatbots to produce easily understandable, medically relevant, culturally appropriate, and empathetic responses to oncology patient queries was a key focus of many of the included studies [19,32,45,46]. While chatbots may be more easily applied to diagnostics, treatment, and monitoring [26], progress on interpersonal-type communications is being made, despite challenges noted on the algorithmic development side [45]. As a sign of how far these chatbots have come, one social media–based study found that nonhuman responses were rated higher on every measure by expert health care evaluators, including empathy and quality, and greatly preferred to the clinician responses [32]. Others further illustrate these applications through immersive and affect-aware interventions, including AI-driven virtual reality (VR) therapy for anxiety and depression [47,48] and sentiment-aware conversational agents designed to detect and respond to emotional distress [49].

In another notable example, the unique emotional needs of teen and young adult patients with cancer were addressed by the delivery of positive psychology skills via the Vivibot chatbot [14]. As this age group typically experiences heightened anxiety and depression posttreatment completion, this kind of intervention fills a previously unmet need [55].

### Caregiver Support

Empathic AI also plays a critical role in supporting caregivers, who often experience high levels of stress and burnout. Unsurprisingly, 52% (23/44) of studies were also concerned with addressing aspects of caregiver emotional support. AI applications were found to offer tailored resources, emotional support, and coping strategies, helping caregivers manage their own well-being while supporting their loved ones through cancer care. For example, AI-based cofacilitators for online cancer support groups were shown to provide targeted emotional support for both patients and their caregivers in response to the detection of emotional distress [16]. Also, as patient care is a complex endeavor involving an interconnected team of clinicians, patients, and caregivers, the use of empathic AI chatbots can be at the center of an integrated and holistic approach to support [26,43,50]. Additional evidence highlights AI-supported environments that indirectly or explicitly assist caregivers through shared decision-making and emotional scaffolding, particularly in advanced cancer contexts [22,34].

Of particular interest is the potential of LLMs as providers of real-time, individually customized, and up-to-date medical information, with their concomitant risks and benefits, to allow for informed, complex, and empathic decision-making among caregivers for oncology patients [30]. A more speculative take on AI technologies in health care argues that the next step may well be social robots imbued with artificial empathy as the caregivers of the future [20]. Ultimately, any of these configurations may be thought of as hybrid intelligent human-AI care systems.

### Palliative and End-of-Life Care

In palliative and end-of-life care settings, empathic AI has been particularly effective in providing compassionate communication and emotional support, with 30% (13/44) of studies referencing the use of AI in these contexts. These systems help both patients and health care providers navigate sensitive conversations, ensuring that care remains patient-centered and aligned with individual values during the final stages of life. For instance, the VR psychological support tool used in the SafeSpace study [51] sought to integrate guided relaxation exercises and compassionate mind training for oncology patients afflicted with a range of cancer types, including those under palliative care. Statistically significant improvements were found in the psychological measures of mood and emotional well-being, with overall reductions in stress levels demonstrated due to the safe and soothing environment simulated by the intervention. Simulated VR travel to memorable places was another unique application of empathic AI that boosted physiological and mental health outcomes, particularly depression and anxiety, for patients with terminal cancer[51]. Other researchers have used NLP approaches, such as analyzing emotion intensity or identifying psychosocial challenges, to address end-of-life discussions with patients with cancer and provide them with targeted resources specific to their stage of the cancer journey [22,24,35,36,41,56].

## Discussion

### Principal Findings

This review demonstrates that empathic AI is emerging as a powerful and transformative force within oncology, with broad clinical applications from education to diagnostics and treatment planning to emotional support. As such, empathic AI has the potential to enhance not only the efficacy and accuracy of care but also its psychosocial dimensions. We found that the majority of studies focused on patient support, with 85% (38/44) addressing tailored patient education and 91% (40/44) addressing patient emotional and psychosocial support. While less frequently represented, a subset of studies also explored applications in diagnostics and decision-making support (19/44, 43%) and palliative and end-of-life care (13/44, 30%), highlighting additional areas where empathic AI is beginning to gain traction. Across domains, evidence illustrates that AI systems are increasingly being used to address long-standing gaps in cancer care delivery. These technologies are reconfiguring traditional models of care into more adaptive, responsive, and patient-centered frameworks.

It is important to distinguish between human empathy and algorithmic or “simulated” empathy. While human empathy arises from lived experience, relational context, and moral judgment, empathic AI operates through pattern recognition, affective cue detection, and response optimization [58]. These systems do not “feel” empathy but rather approximate empathic behaviors through language, timing, and contextual sensitivity. This positions empathic AI to be understood not as a substitute for human connection but as a decision support and interaction support that may enhance communication, accessibility, and emotional scaffolding within clinician-led care.

A consistent theme throughout the examined literature is the redefinition of what it means to deliver holistic care. Empathic AI enables care that integrates clinical precision with personalized emotional support, bridging a gap that has long challenged providers operating under time and resource constraints. This convergence is particularly critical in oncology, where patients face complex treatment pathways intertwined with profound emotional and existential burdens. Across studies, LLMs and chatbot technologies were used to adapt educational content to user-specific contexts, including cancer type, treatment stage, emotional state, and cultural or linguistic preferences.

To bring these capabilities into everyday practice, clinicians can adopt empathic AI in targeted, workflow-compatible ways. For example, LLMs can be embedded into electronic health record systems to generate on-demand phrasing suggestions for emotionally sensitive conversations, such as treatment failure or end-of-life planning. Moreover, AI cofacilitators can personalize group therapy or education sessions in real time by responding to emotional cues. Additionally, sentiment analyses or AI-generated chat summaries can be used to brief providers ahead of visits, helping them tailor communication strategies to patient needs. AI’s ability to contextualize medical information, detect distress, and respond with calibrated empathy suggests that these systems can complement and bolster human care, particularly in emotionally charged settings such as diagnosis disclosure, palliative care, and end-of-life decision-making.

Evidence from this review further suggests that empathic performance in digital oncology settings is not solely a function of model sophistication but of interactional design and workflow integration. Studies measuring cancer-related live chat environments and simulation-based systems highlight that digital clinical empathy requires intentional strategies (ie, timely acknowledgment, authenticity, appropriate boundary setting, and contextual responsiveness) and may be best sustained through hybrid models that combine AI-enabled support with human oversight [34-36].

Moreover, the high levels of user satisfaction reported in interventions such as Vivibot [14], Vik [17], Rosa [18], and social media–based studies [19,32,57] underscore that patients are receptive to receiving education, treatment planning, and emotional support from AI systems. These findings raise important implications for the clinical role of AI in extending the reach and impact of human oncology care, rather than simply imitating it. Rather than merely mimicking human interaction, empathic AI can help scale patient-centered care by offering timely, emotionally calibrated support, especially in scenarios where time constraints or emotional fatigue may limit provider capacity. Notably, findings from this review suggest that patients may sometimes prefer AI-generated responses over those from physicians when it comes to clarity, empathy, and accessibility [19,29,32]. Such findings challenge traditional assumptions about the limitations of machine-delivered care and suggest a potential reimagining of the therapeutic relationship. In oncology, clarity, emotional tone, and trust are particularly sensitive. For clinicians, this emerging preference signals an opportunity to align care delivery models with patient communication expectations. AI can serve as a buffer, or even a bridge, between emotionally overwhelmed patients and time-constrained providers. Empathic AI-mediated tools may help close gaps in emotional availability, ensure message consistency, and reduce the interpersonal strain that often accompanies sensitive care discussions.

Empathic AI also holds potential to reshape the patient-to-provider relationship. For patients, AI expands access to personalized education and emotional support, democratizing information and empowering users to take an active role in their treatment. This is especially significant for historically underserved populations or individuals with low health literacy, who may benefit most from scalable, emotionally attuned technologies. However, emerging evidence indicates that patient engagement with empathic AI tools is strongly shaped by trust, digital literacy, perceived usefulness, privacy concerns, and the extent to which AI systems are meaningfully connected to clinicians and care pathways. This highlights implementation success dependent as much on sociotechnical context as on model performance [25].

From a provider perspective, AI serves as both a diagnostic and treatment decision support system and a partner in empathic communication. AI-generated conversation summaries, real-time feedback, and emotion-sensitive prompts can enhance provider-patient dialogues and reduce burnout by offloading cognitive and emotional labor, such as the analysis of patient decision-making and emotions provided by the PRIME-2 (Patient Reported Information Multidimensional Exploration version 2) framework [46]. However, these benefits come with caution. The risk of overreliance on AI-generated outputs and the potential erosion of clinicians’ empathic instincts raise critical concerns about long-term implications for professional development and patient trust.

Despite the promise of empathic AI, several challenges must be addressed before these systems can be safely and effectively deployed in clinical settings. The question of algorithmic empathy remains unresolved. Evidence suggests that generative AI responses to cancer-related decision-making scenarios may vary by patient demographics and clinical framing, with inconsistencies in tone, content, and guidance that could differentially influence patient perceptions and treatment decisions, underscoring the need for careful validation and health equity–focused oversight [22,24]. While AI can simulate or mimic affective responses and detect emotional cues, true empathic understanding rooted in shared experience remains uniquely human. As such, AI should be positioned as a decision support tool, not as a replacement for traditional, physician-led care. Overreliance on AI in emotionally sensitive encounters could lead to depersonalization or erosion of therapeutic rapport between patient and provider, particularly if patients perceive machine-generated responses as inauthentic or misaligned with their values.

Models trained on nonrepresentative data may also risk reinforcing racial, cultural, and linguistic biases, leading to emotionally inappropriate or even harmful interactions. In oncology, where communication must be attuned to cultural norms, family dynamics, and existential concerns, these missteps could carry particularly high stakes. Specifically, loss of trust in providers and institutions is detrimental to cancer care, as trust remains the foundation of health care in sensitive and difficult situations and among vulnerable populations. Safety is further complicated by the risk of misinformation or emotionally manipulative outputs. Moreover, sociocultural dynamics shape both the reception and use of empathic AI. Concepts of empathy, emotional expression, and caregiving may vary considerably across cultures; what is perceived as compassionate in one context may be viewed as intrusive or inappropriate in another. Developers must engage patients, caregivers, and clinicians from diverse backgrounds in early design to ensure cultural alignment and application for all populations.

Ensuring that AI systems provide reliable, culturally sensitive, and context-appropriate support will require robust algorithmic guardrails, clinician oversight, and rigorous validation. Without these safeguards, empathic AI may inadvertently exacerbate existing health disparities among marginalized populations already facing barriers to emotionally supportive care.

The findings of this review underscore the value of hybrid clinical care models, wherein humans and machines complement each other, termed human-AI symbiosis [39]. While empathic AI systems show promise in extending the reach of clinicians, personalizing education, and offering emotional scaffolding for patients and caregivers, their successful integration into clinical workflows remains dependent on several critical factors. Effective implementation will depend on careful design, robust training data, and a commitment to maintaining the human essence of medicine. Given the rapid pace of model development and evolving user expectations, adoption should proceed cautiously, with a focus on adaptability and long-term clinical usefulness. Moving forward, research must explore the longitudinal impacts of empathic AI interventions, develop integration frameworks grounded in ethical and cultural humility, and assess performance across diverse populations and care settings. Interdisciplinary collaboration will be essential to ensure that future systems are not only intelligent and accurate but also trustworthy, equitable, compassionate, and responsive to real-world clinical needs. To support practical interpretation, Table 2 summarizes key challenges associated with empathic AI in oncology alongside potential mitigation strategies identified across the included literature.

**Table 2.** Challenges, risks, and mitigation strategies for empathic artificial intelligence in cancer care.

Challenge	Description	Potential mitigation strategies
Algorithmic empathy versus human empathy	Empathic AI^a^ systems simulate empathic behaviors through pattern recognition and affective cue detection but lack lived experience, moral reasoning, and relational accountability. This raises concerns about authenticity, depth, and appropriateness of emotional responses in sensitive oncology contexts.	Position empathic AI as decision support and interaction support rather than replacement for human care; embed clinician oversight and escalation pathways; and explicitly communicate AI’s role and limitations to patients to preserve trust and relational integrity.
Reliability, trust, and interpretive risk	Empathic AI-generated responses may vary in tone, emotional sensitivity, or guidance depending on prompt structure, clinical framing, or patient demographics, potentially shaping patient perceptions, fostering over trust or skepticism, and influencing clinical decision-making.	Implement algorithmic guardrails and standardized response calibration; conduct bias and consistency audits across subgroups; intentionally design user interfaces to reinforce clinician accountability; use scenario-based validation prior to deployment in emotionally sensitive use cases; and provide clinician training on appropriate AI use.
Misinformation risk	Generative AI systems may produce fluent, confident, but clinically incorrect or incomplete responses, which can be particularly harmful in oncology decision-making and end-of-life contexts.	Maintain human-in-the-loop validation for high-stakes outputs; restrict autonomous response generation in diagnostic and treatment contexts; and integrate citation requirements, uncertainty signaling, and clinician review mechanisms.
Equity, bias, and cultural alignment	Training data may encode structural inequities or fail to account for cultural variation in emotional expression, caregiving norms, and communication styles, resulting in emotionally inappropriate or inequitable interactions.	Use participatory and co-design approaches with diverse patients, caregivers, and clinicians; prioritize inclusive datasets; localize language, tone, and interaction styles; and conduct ongoing health equity and subgroup performance audits.
Privacy, data governance, and emotional data sensitivity	Empathic AI systems often process highly sensitive emotional, psychosocial, and behavioral data, increasing risks related to privacy, consent, and secondary data use.	Establish robust data governance frameworks; apply privacy-by-design principles; ensure secure infrastructure and role-based access controls; and clarify consent processes for emotional and conversational data use.
Workflow integration and clinician burden	Poorly integrated AI tools may increase cognitive load, disrupt clinical workflows, or generate alert fatigue, undermining adoption and trust.	Design workflow-compatible tools aligned with clinical routines; pilot implementations in real-world settings; and iteratively refine systems based on clinician feedback and usability testing.

a AI: artificial intelligence.

### Strengths and Limitations

This review offers a comprehensive analysis of empathic AI in cancer care, yet several limitations should be noted. First, this review included studies available only in English language, which may limit the generalizability of findings to global health care contexts. Cultural variations in emotional expression, communication styles, and attitudes toward AI are likely to influence both the development and reception of empathic AI systems. Furthermore, although a snowballing technique was used to enhance inclusivity, the reliance on peer-reviewed literature indexed in PubMed may have excluded relevant studies published in interdisciplinary journals, conference proceedings, or gray literature, particularly those from fields such as human-computer interaction or behavioral science.

Another limitation relates to the rapidly evolving nature of AI technologies. Given the pace of advancement in the field, some of the models and interventions included in this review may already be outdated or replaced by more sophisticated systems. While this pace of innovation reflects momentum in the field, it also complicates assessments of clinical readiness. Current tools may not yet meet the usability, safety, or integration standards required for large-scale adoption, underscoring the need for ongoing evaluation in real-world settings.

However, this fast-paced innovation can also be viewed as a strength, as the continual refinement of AI models suggests that future iterations will likely be more accurate, more empathetic, and better integrated into clinical workflows. By capturing a broad timeline of development from 2018 to early 2025, this review not only reflects the field’s foundational growth but also establishes a baseline against which future progress can be measured.

It is also important to note that included studies were methodologically heterogeneous, encompassing diverse designs, populations, interventions, and outcome measures. The absence of standardized metrics for evaluating empathic performance limits direct comparison across studies, although this heterogeneity reflects the early and exploratory nature of the field.

Additionally, there was no standardized metric for evaluating “empathy” in AI-generated interactions across studies. This inconsistency complicates efforts to compare effectiveness across interventions. Some studies used patient satisfaction or emotional resonance as proxies, while others relied on subjective expert evaluation. At the same time, emerging work in sentiment modeling and empathic conversational agent development suggests early methodological movement toward quantifying affective signals, although approaches remain heterogeneous and not yet clinically standardized [49,53,54]. Future research should focus on a shared operational definition of empathic AI performance to establish clear benchmarks for clinical implementation.

### Public Health Implications

With careful design and oversight, empathic AI has the potential to advance public health by expanding access to emotionally responsive care and addressing structural gaps in patient-provider communication, trust, and psychosocial support. These technologies could help bridge long-standing inequities by reaching historically underserved populations facing linguistic, geographic, or cultural barriers to care. In resource-limited settings, empathic AI may also reduce clinician burden, promote continuity of care, and strengthen system resilience by extending the reach of essential health services.

In oncology, empathic AI platforms could deliver timely, culturally sensitive, and emotionally attuned support to help mitigate disparities in diagnosis, treatment adherence, and survivorship. By enhancing provider communication and tailoring care to diverse patient needs, these systems can counteract the effects of systemic bias and institutional mistrust. Integration into care settings may also enable early detection of patient distress and improve referral pathways to behavioral health and palliative services, offering a scalable means of bridging mental and physical care.

Implementation of empathic AI in low-resource or global health contexts may offer affordable, scalable strategies to support patient-centered cancer care where human capital is limited. As digital health infrastructure continues to evolve, empathic AI should be recognized not only as a clinical innovation but as a system-level tool for strengthening communication-centered models of care that foster patient engagement, empowerment, and trust. By capturing data on emotional needs, communication gaps, and engagement patterns, these tools may also inform more responsive, equity-driven public health surveillance and planning.

### Conclusions

Empathic AI holds immense promise to revolutionize cancer care by enabling a more patient-centered, holistic, and emotionally sensitive approach. Oncology presents distinct emotional and ethical challenges, making it a uniquely high-stakes setting for the application of empathic AI. With thoughtful development and implementation, these tools can help close communication gaps, improve diagnostics and treatment decision-making, and support the emotional well-being of both patients and caregivers.

The creation of empathic AI–driven portals for patients and providers, for example, can improve engagement and satisfaction, expand access to culturally tailored education, and personalize treatment planning. These tools can and should be applied across the oncologic care continuum from identifying at-risk patients, to supporting treatment navigation and recovery, to helping patients and caregivers cope with the emotional burden of a cancer diagnosis. While this review highlights early promise and high user receptivity, clinical integration must be guided by rigorous validation, ethical oversight, and a deep understanding of real-world workflows and health system readiness.

Empathic AI should be viewed as an adjunct to human-centered care, not as a replacement for current practices and procedures. Clinicians must lead its implementation to ensure that empathy, trust, and clinical excellence remain central to oncologic practice. To support responsible adoption, health care systems should proactively invest in the infrastructure required to evaluate and scale these tools. Modular pilot programs such as leveraging LLMs to coauthor patient education materials, deploying chatbot cofacilitators in support groups, or using NLP tools to identify patients in emotional distress can help pave the way to widespread implementation. Other practical applications include automated phrasing assistance in clinical documentation, simulation-based training for difficult conversations, and tools to detect and mitigate caregiver burnout.

This review highlights the distinct value and complexity of applying empathic AI in oncology, where sensitivities differ markedly from other clinical contexts. The findings underscore the need for an oncology-specific framework that identifies key emotional touchpoints, proposes clinical use cases, and integrates safeguards to mitigate ethical and psychosocial risks. With continued advancement, empathic AI can help foster a health care environment that is both emotionally attuned and operationally efficient—particularly in cancer care, where emotional resonance and communication quality are as vital as clinical accuracy. The path forward requires rigorous validation, interdisciplinary collaboration, and an unwavering commitment to equity and patient voice. As we move toward the next generation of cancer care, the thoughtful integration of empathic AI offers an unprecedented opportunity to reimagine how we deliver care.

## Supplementary material

10.2196/82336 Multimedia Appendix 1 Summary table representing the characteristics of selected studies (revision).

10.2196/82336 Checklist 1 PRISMA-ScR checklist.

## Abbreviations

AI: artificial intelligence
LLM: large language model
ML: machine learning
NLP: natural language processing
PRIME-2: Patient Reported Information Multidimensional Exploration version 2
PRISMA: Preferred Reporting Items for Systematic Reviews and Meta-Analyses
VR: virtual reality
