# Supplementary material for: Empathic AI for Patient-Centered Cancer Care: A Scoping Review of Patient Navigation, Support, and Clinical Practice
Source: JMIR Cancer. 2026 Apr 9;12:e82336. doi: 10.2196/82336 (PMC13065233; doi:10.2196/82336)
Supplement: Multimedia Appendix 1 [file cancer-v12-e82336-s001.docx]

Multimedia Appendix 1

Table S1. Summary table representing the characteristics of selected studies.

| First author, year | Purpose | Study type | Utility of Empathic AI in Cancer Care | Author’s conclusion |
| --- | --- | --- | --- | --- |
| Greer et al (2019) [14] | To examine the feasibility of delivering positive psychology skills via the Vivibot chatbot and its effects on key psychosocial well-being outcomes in young adults treated for cancer. | Randomized Control Trial | Patient Emotional Support, Caregiver Support, Care Plan Optimization | Conclusions suggest that the chatbot format provides a useful and acceptable way of delivering positive psychology skills to young adults who have undergone cancer treatment and supports anxiety reduction. |
| Leung et al (2024) [15] | To develop and evaluate the Artificial Intelligence–based Co-Facilitator (AICF), an innovative tool designed to monitor group cohesion in web-based cancer support groups (OSGs). Group cohesion is vital for the emotional well-being and connection of participants, but it can be hard to assess in text-based environments where nonverbal cues are absent. The purpose of the study was to see if AICF could effectively detect shifts in group dynamics and provide real-time feedback to therapists, ensuring that empathy and support are always present. This would help support the emotional needs of cancer patients, strengthening therapeutic experience in online settings. | Single Arm Trial | Tailored Patient Education, Tailored Provider Education, Patient Emotional Support | Findings suggest AICF can successfully detect group cohesion in online cancer support groups, showing its potential as a valuable tool for therapists. By combining machine learning algorithms with human insight, AICF can help therapists recognize when group cohesion is faltering and when real-time intervention is necessary. This is especially important in web-based settings, where nonverbal cues and in-person interaction are missing. The ability to monitor and support emotional well-being in these groups helps ensure that patients feel heard, validated, and connected. AICF has the potential to make online support groups a more empathetic and responsive environment for cancer patients. |
| Leung et al (2023) [16] | To provide an overview and understanding of the feasibility, acceptability, validity, and reliability of an Artificial Intelligence-based Co-Facilitator (AICF) within online cancer support groups. Specifically, the study aimed to assess how AICF could monitor distress levels in real time by analyzing text-based interactions during group sessions. | Mixed-Methods Single Arm Trial | Patient Emotional Support, Caregiver Support, Tailored Patient Education, Tailored Provider Education, Care Plan Optimization, Palliative and End-of-Life Care | Conclusions suggest while quantitative results showed that AICF demonstrated only some validity in detecting distress levels, the qualitative findings indicated that AICF was highly valued for its ability to identify real-time distress issues and allow therapists to provide proactive, individualized support. This real-time detection allowed for better timely interventions, ensuring that group members received the emotional support they needed, especially when facing heightened distress. However, therapists expressed concerns about the ethical implications of AI-driven distress detection, particularly regarding privacy and liability. Despite these concerns, the acceptability of AICF among therapists was generally positive, suggesting it could play a valuable role in enhancing therapist-patient empathy and emotional engagement in virtual support groups. |
| Chaix (2019) [17] | To design a chatbot (Vik) to empower patients with breast cancer and their relatives. To evaluate one year of conversations between patients with breast cancer and a chatbot. | Pilot Study | Diagnostic, Tailored Patient Education, Tailored Provider Education, Patient Emotional Support, Caregiver Support, Care Plan Optimization | Findings demonstrate that it is possible to obtain support through a chatbot given that the chatbot in the current study improved the medication adherence rate of patients with breast cancer. The overall satisfaction of users was 93.95%, and 88.00% of users said that the chatbot provided them with support and helped them track their treatment effectively. |
| Siglen et al (2022) [18] | To develop a pilot version of an app (Rosa) that can perform digital conversations with breast or ovarian cancer patients about genetic BRCA testing, using chatbot technology, to identify best practices for future patient-focused chatbots. | Pilot Study | Diagnostic, Tailored Patient Education, Tailored Provider Education, Care Plan Optimization | Findings reveal that despite the limited AI functionality of the chatbot, users liked the layout and found the chatbot trustworthy and reader friendly. Although building chatbot is challenging, expensive, and time-consuming, users had a positive attitude to the chatbot and would use it in a real life setting if given to them by healthcare personnel. |
| Chen et al (2024) [19] | To evaluate the ability of AI chatbots (GPT-3.5, GPT-4, and Claude AI) to generate high-quality, empathetic, and readable responses to cancer-related questions posed by patients on social media. The comparison was made against responses from oncologists to determine how well AI could replicate human-like, compassionate, and clear communication in the context of cancer care. | Equivalence trial | Tailored Patient Education, Patient Emotional Support | The study found that AI chatbots (particularly Claude AI) produced responses that were significantly better in terms of quality, empathy, and readability compared to oncologists' responses. The Flesch-Kincaid Grade Level indicated that chatbot responses were more complex than those of oncologists but still comparable in terms of readability. The findings suggest that AI chatbots can generate responses that are effective, empathetic, and comprehensible, making them a potential tool for improving patient communication in oncology. Further research is necessary to understand how chatbot-facilitated interactions can be integrated into clinical practice and their impact on patient outcomes. |
| Morrow et al (2023) [20] | To explore the relationship between artificial intelligence (AI) technologies and compassion in healthcare. The review seeks to provide a comprehensive and balanced perspective on how AI technologies can enhance compassionate care, identify gaps in current knowledge, and highlight key areas where AI could support compassion in healthcare research and practice. | Systematic Scoping Review | Diagnostic, Tailored Patient Education, Tailored Provider Education, Patient Emotional Support, Caregiver Support, Care Plan Optimization | The review concludes that there is an association between AI technologies and compassion in healthcare, with growing worldwide interest over the past decade. AI technologies are being utilized in many healthcare contexts to enhance empathetic awareness and response, relational behavior, communication skills, health coaching and education, therapeutic interventions, clinical assessment, and to offer health information and advice. The findings suggest a reconceptualization of compassion as a human-AI intelligent caring system that that functions at both individual and healthcare system levels to understand and alleviate various types of suffering. |
| McDarby et al (2024) [21] | This study aims to examine how ChatGPT responds to a hypothetical patient decision-making question in advanced cancer and whether responses vary based on patient demographic and clinical characteristics. | Cross-sectional Study | Tailored Patient Education, Patient Emotional Support, Caregiver Support, Care Plan Optimization, Palliative and End-of-Life Care | Conclusions highlight that ChatGPT responses to advanced cancer decision-making questions vary by patient demographics and clinical context, suggesting that the tone and content of AI-generated guidance, potentially including empathic elements, are inconsistent and may influence patient treatment decisions. |
| Jo et al (2024) [22] | This study aims to evaluate the ability of generative AI tools (GPT-4, Google Bard, and CLOVA X) to accurately and effectively answer patient questions about colorectal cancer. | Cross-sectional Study | Diagnostic, Tailored Patient Education, Tailored Provider Education, Patient Emotional Support, Care Plan Optimization | Conclusions suggest that generative AI tools demonstrate communicative and emotional competence comparable to or better than traditional patient education materials, supporting their potential role in patient-centered information delivery. |
| Clerici et al (2024) [23] | This study aims to evaluate the performance and sensitivity of commonly used chatbots in providing information about a rare pediatric cancer (rhabdomyosarcoma) as assessed by clinical experts. | Cross-sectional Study | Diagnostic, Tailored Patient Education, Patient Emotional Support, Palliative and End-of-Life Care | Conclusions highlight that while chatbots provide accurate general cancer information, they lack sensitivity and emotional responsiveness when addressing treatment and referral needs, underscoring current limitations in empathic AI support for young cancer patients. |
| Alanezi (2024) [24] | This study aims to investigate factors influencing patients’ engagement with ChatGPT for accessing health-related information. | Prospective Cohort Study | Tailored Patient Education, Patient Emotional Support | This study identified 28 influencing factors that may affect patients’ engagement with ChatGPT. Lack of trust, lack of skills and competencies among users, technical limitations, poor information quality, and ethical concerns were identified to negatively affect patients’ engagement. ChatGPT was identified to be effective in positively contributing to user-related factors like perceived ease of use, perceived usefulness, and satisfaction. Major issues were associated with the application and its features such as technical limitations in integration, poor connectivity with healthcare professionals, and ethical concerns including privacy and security, legal regulations, and standards of practice. While ChatGPT has undoubtedly achieved users’ interest in its features and functionalities, there are still some concerns which may limit patients’ engagement. |
| Xu et al (2021) [25] | To review recent trends and advances in chatbot use in medicine, particularly in the context of cancer therapy. Patient emotional support and counseling are examined, as are diagnostics, treatment, monitoring, improving workflows, and the promotion health-oriented behaviors. Drawbacks of using chatbot technology in healthcare are also explored and include ethical, security, regulatory, and evaluation challenges that also impede their uptake. | Systematic Review | Diagnostic, Tailored Patient Education, Tailored Provider Education, Patient Emotional Support, Caregiver Support, Care Plan Optimization | Conclusion highlights that there is much room left to explore the potential impact of chatbots on cancer diagnostics, treatment, monitoring, and emotional support. Rapid iteration of the technology will continue to pose challenges in terms of usability, suitability, and evaluation. Human medical providers should remain at the forefront while deploying chatbots in a targeted, careful, and well-vetted way in healthcare to maximize their benefits and minimize their risks. |
| Goumas et al (2024) [26] | To illustrate the types, the advantages and disadvantages, and the challenges of the novel chatbots in medicine and particularly in oncology. Furthermore, an expert opinion about this topic is thoroughly discussed. This review evaluates the advantages and disadvantages of these chatbots and reveals their challenges and obstacles for medicine and cancer. | Narrative Review | Diagnostic, Tailored Patient Education, Tailored Provider Education, Patient Emotional Support, Caregiver Support, Care Plan Optimization | Conclusions suggest that AI technology and novel chatbots have remarkably revolutionized modern life, particularly in the field of medicine. Despite the fact that such technologies have various advantages, there exist disadvantages too, and important challenges and obstacles have arisen, especially regarding their use in cancer cases. Patients and physicians should be informed about chatbot technology and use it only in a few specific and safe situations when the benefits outweigh the risks. |
| Turner (2023) [27] | To examine the question: “Can a person trust an inhumane amoral agent, such as a large language model artificial intelligence (AI) chatbot, to manifest the goodwill and willingness normally required in order for it to be deemed trustworthy?” This article explores the relationship between the cancer patient, their physician, and AI chatbot in a proposed tripartite, consultative, personalized approach to shared care in precision molecular oncology. | Editorial | Tailored Patient Education, Patient Emotional Support, Care Plan Optimization | This editorial explores the nature of trust between human agents and machines. It also contemplates AI-enhanced technical precision in state-of-the-art cancer management, complemented by trustworthy, holistic clinical care by a physician, for each individual patient. |
| Alanzi et al (2023) [28] | To assess the public's awareness of the link between obesity and cancer risk in central Saudi Arabia, while also evaluating the feasibility of using ChatGPT as an empathetic educational tool to enhance public understanding. The study aimed to explore whether AI-based interventions like ChatGPT could provide personalized, compassionate support to individuals in understanding the gravity of obesity’s connection to cancer, while also offering an empathetic, approachable way to learn about the issue. | Mixed-Methods Cross-sectional Study | Diagnostic, Tailored Patient Education, Tailored Provider Education, Patient Emotional Support, Caregiver Support | Conclusion suggests that 65% of participants were not fully aware of the link between obesity and cancer, but it also highlighted ChatGPT's potential to serve as a compassionate, cost-effective tool for improving public awareness. Participants identified the AI’s ability to provide personalized, non-judgmental, and empathetic information as an asset, helping individuals feel more comfortable with the sensitive topic of obesity and cancer. However, challenges like information inaccuracy and concerns about emotional intelligence were noted. Despite these, ChatGPT’s ability to offer accessible and empathetic education was viewed as a promising tool to address the knowledge gap in Saudi Arabia regarding obesity-related cancer risks. |
| Koranteng et al (2024) [29] | To evaluate the ethical implications of deploying artificial empathy (AI that mimics compassion) in oncology. It examines the potential risks and benefits of integrating AI-driven empathetic responses into cancer care, particularly in the oncologist-patient relationship, which relies heavily on genuine emotional understanding. The authors question whether AI can effectively replicate human empathy and what the ethical concerns might be in replacing human clinicians with machines for emotional support. | Op-Ed, Viewpoint | Tailored Patient Education, Patient Emotional Support, Caregiver Support | Findings suggest artificial empathy holds the potential to assist oncology care by providing emotional support during diagnosis, treatment, and survivorship, while reducing clinician burnout caused by compassion fatigue. However, there are ethical risks associated with relying on machines for empathy. These include the reduction of true human empathy in care, furthering healthcare inequities, and alienating patients who may feel disconnected from the genuine emotional understanding of human clinicians. While AI might be acceptable in some situations, a careful balance is necessary, ensuring that patients are aware when interacting with AI and that the technology complements rather than replaces human care. |
| Sezgin et al (2025) [30] | To investigate the use of large language model-supported tools in providing empathic, accurate, and reliable information to caregivers of pediatric cancer patients, aiming to improve their understanding and emotional support. | Cross‐sectional study observational study | Caregiver Support, Tailed Patient Education, Tailored Provider Education | Conclusions suggest that LLM-supported tools can effectively enhance caregivers' knowledge of pediatric oncology, offering not only accurate information but also an empathetic approach tailored to the unique emotional needs of caregivers. |
| Leung et al (2021) [31] | To evaluate the development and performance of an empathic AI-based cofacilitator for tracking emotional distress, participant engagement, and group cohesion in online cancer support groups, aiming to provide personalized, real-time emotional support for participants. | Quasi-experimental, developmental study utilizing mixed methods | Patient Emotional Support, Caregiver Support, Tailed Patient Education, Care Plan Optimization | Conclusions suggest that an AI-based cofacilitator has the potential to enhance real-time emotional support and engagement in online cancer support groups, providing a personalized, empathic approach to addressing participants' emotional distress and individual needs. |
| Ayers et al (2023) [32] | To determine whether an AI chatbot assistant can respond to written patient questions in a way that shows quality and empathy on a comparable level to that of physicians. A social media forum (Reddit) was used to source medical questions from the public, and the written responses of clinicians and chatbots were rated by expert healthcare evaluators. | Cross-sectional Study | Tailored Patient Education, Patient Emotional Support | The study found that chatbot responses were longer, more substantive, and heavily preferred over the physician responses. They were consistently rated higher on empathy and quality. As such, AI assistants might be considered for incorporation into clinician messaging workflows with patients. As time constraints frequently lead physicians to communicate in a terse and less empathetic way, AI assistance could help with drafts that can be reviewed and modified. High-quality responses may improve numerous health-related behaviors and overall patient outcomes, all while reducing physician burnout. |
| De Silva et al (2018) [33] | To design and implement a machine learning and natural language processing (NLP) framework to analyze patient interactions within online support groups (OSGs) in the context of cancer care. This analysis focused on identifying patient-reported behaviors, emotions, treatment decisions, and side effects, with the goal of providing empathetic insights into patient decision-making. By using prostate cancer as a model, the study aimed to examine how online social influences and emotional expressions shaped patient decisions from diagnosis through recovery. This framework emphasizes the importance of empathetic support within OSGs, ensuring that patient voices are heard and understood. | Cross-sectional Study | Diagnostic, Tailored Patient Education, Tailored Provider Education, Patient Emotional Support, Caregiver Support | Conclusions highlight the significant role that online support groups play in cancer care, especially through the lens of empathetic decision-making and emotional support. By using AI-driven insights, healthcare providers can better understand how patients engage with information and emotional support. The study identifies three distinct decision-making groups (Paternalistic, Autonomous, and Shared) and tracks the emotional dynamics involved in their decisions. Furthermore, it illustrates how patients evolve within the OSGs, transitioning from seeking support to providing it, creating a cycle of empathetic mutual care.  The ability of the AI framework to analyze these emotional and decision-making behaviors ensures that healthcare providers can adopt a more empathetic approach to care, meeting individual patient needs more effectively. Understanding patients’ emotional cues within their support group conversations allows for more compassionate care planning. |
| Lanfer et al (2024) [34] | This study aims to explore and evaluate techniques of digital clinical empathy in a familial cancer live-chat setting, focusing on how health professionals understand, communicate, and act upon users’ emotional perspectives. | Experience-based codesign (EBCD) and mixed methods | Tailored Patient Education, Tailored Provider Education, Patient Emotional Support, Caregiver Support, Care Plan Optimization | Conclusions highlight that digital clinical empathy in cancer-focused live chats requires intentional strategies to convey understanding, emotional support, and authenticity, and that hybrid models combining AI and human professionals may help sustain empathic patient communication. |
| Kane et al (2024) [35] | This study aims to develop and evaluate a dialogue management framework (SOPHIE) that simulates a virtual cancer patient to help physicians practice empathy and end-of-life communication skills. | Mixed-Methods Evaluation | Tailored Patient Education  Tailored Provider Education  Patient Emotional Support  Palliative and End-of-Life Care | This paper introduced SOPHIE, a flexible schema-guided dialogue framework designed as a virtual standardized cancer patient to help doctors practice patient conversations. Evaluations from a pilot experiment showed that SOPHIE was fluent, natural, and emotionally appropriate, outperforming a neural baseline model. However, limitations in user understanding were identified, prompting future work to enhance semantic interpretation and develop improved response generation strategies. |
| Ali et al (2023) [36] | This study aims to describe the participatory design and initial evaluation of SOPHIE, a virtual patient system for training physicians in sensitive patient-physician communication. | Iterative participatory; Pilot study | Tailored Patient Education  Tailored Provider Education  Patient Emotional Support  Caregiver Support  Palliative and End-of-Life Care | This study explored early findings from multi-stage research focused on improving patient-physician communication, particularly in conversations between terminal cancer patients and oncologists. It highlighted effective traits such as asking questions, avoiding lecturing, and delivering news positively, alongside the development of SOPHIE, an online standardized training system using avatars to simulate patient interactions. SOPHIE-like systems have the potential to address gaps in palliative care training globally, especially in low- and middle-income countries where access to quality comfort care remains limited. |
| Goisauf and Cano Abadía (2022) [37] | To evaluate the current state of AI ethics in radiology through a philosophical and social science lens. The analysis focuses on what types of ethical issues AI poses in biomedical research and how these topics are being explored in the context of radiology, and specifically breast cancer. With a view to avoiding the harmful consequences of its application, guiding principles and expert recommendations are examined. | Systematic Review | Diagnostic, Tailored Patient Education, Tailored Provider Education, Patient Emotional Support, Care Plan Optimization | A lookback over literature concerned with ethical and socioeconomic issues related to AI use in radiology discovered that it lacked depth with regard to societal values and harbored bias with relation to breast cancer patient characteristics. The discourse was often framed as a technical problem with a technical solution. Transparency and trust were highlighted as oft-used terms that were not clearly defined. The authors suggest that interdisciplinary research among social and clinical scientists can help clarify language and increase patient engagement and support. |
| Mundinger and Mundinger (2024) [38] | To examine the current state of artificial intelligence (AI) in senology (the study of breast health) and explore its potential future applications. The authors review AI’s impact on breast screening programs, particularly through image analysis and natural language programs, and consider the ethical, regulatory, and practical challenges in advancing AI technology in healthcare. The article underscores the importance of combining AI with human wisdom, empathy, and affection to ensure safe, ethical, and effective use in medical practice | Narrative Review | Diagnostic, Tailored Patient Education, Patient Emotional Support, Care Plan Optimization, Palliative and End-of-Life Care | Conclusions suggest while AI is still in the early stages of its evolution in senology, it holds significant promise for improving breast cancer screening and diagnosis. AI can enhance efficiency by performing routine tasks faster and with fewer errors, which could ultimately improve patient outcomes. However, there are concerns about stability, cybersecurity, liability, and transparency that need to be addressed for AI systems to be safely integrated into healthcare. The authors stress the need for responsible training of AI systems, utilizing meaningful data and real-world studies to assess performance. They emphasize that AI should be combined with human empathy and wisdom to create a more effective, patient-centered approach to healthcare, with a focus on careful regulation to mitigate risks. |
| Turner (2024) [39] | Proposes a new paradigm in theranostic cancer care by integrating artificial intelligence (AI) with physician expertise. The goal is to transform nuclear oncology practice, specifically in the treatment of prostate cancer and neuroendocrine tumors, by combining AI’s data analytics with the physician’s clinical expertise, empathy, and patient care. This approach aims to provide personalized treatment through AI-supported, quantitative data collection and therapeutic dose prescription, overcoming the limitations of the current "one-size-fits-all" approach in radioligand therapy. By fostering a symbiotic relationship between human expertise and AI, the study envisions a more accurate, individualized, and empathetic care model. | Theoretical Conceptual Proposal | Diagnostic, Tailored Patient Education, Patient Emotional Support, Care Plan Optimization, Palliative and End-of-Life Care | Conclusions suggest that human-AI interaction can redefine cancer care by facilitating more personalized and precise theranostics. By leveraging AI’s ability to handle large-scale data analysis and radiomics, along with the physician’s clinical judgment and empathy, the proposed model enables a synergistic relationship that would improve the effectiveness of radioligand therapies. This model envisions improved treatment planning, minimized toxicity, and enhanced patient-physician trust through a more personalized, data-driven, and empathic approach to cancer care. Ultimately, the integration of AI in theranostic reporting will support the moral responsibility of the physician while providing better outcomes for patients. |
| Rogasch et al (2023) [40] | This study aims to evaluate whether ChatGPT can accurately and empathetically answer patient questions and interpret PET/CT imaging reports in clinical settings. | Observational Study | Diagnostic, Tailored Patient Education, Tailored Provider Education, Care Plan Optimization | This study concludes that ChatGPT may offer an adequate substitute for informational counseling to patients in lieu of that provided by nuclear medicine staff in the setting of PET/CT for Hodgkin lymphoma or lung cancer. With ever-decreasing time available for communication between staff and patients, readily accessible AI tools might provide a valuable means of improving patient involvement, the quality of patient preparation, and the patient’s understanding of nuclear medicine reports. Improving the predictability and consistency of ChatGPT would further increase reliability. |
| ^41^ Wei et al (2024) [52] | This study aims to comprehensively review and summarize recent developments in the application of artificial intelligence for the diagnosis, treatment, prognosis, and emotional assessment of patients with peritoneal carcinomatosis. | Systematic Review | Diagnostic, Care Plan Optimization | This review concludes that the newly developed deep neural networks and machine learning algorithms of AI are generally applied in the diagnosis, recurrence assessment, and treatment of peritoneal carcinomatosis (PC). However, the application of AI in PC still has some problems, such as the immature technology, the high cost, and the controversies in moral and ethical direction. In the future, more prospective and rigorous studies are needed to apply AI in PC more systematically and normatively. Research using machine learning methods to select treatment strategies is needed so that clinicians can better individualize treatment regimens to maximize survival benefits. |
| Gliwska et al (2023) [41] | To evaluate the use of natural language processing for analyzing emotion intensity, body image sentiment, and potential psychological difficulties in patients with head and neck cancers, with the aim of supporting cancer care through empathic AI. | Cross-sectional Study | Diagnostic, Patient Emotional Support, Caregiver Support, Tailored Patient Education, Care Plan Optimization, Palliative and End-of-Life Care | Conclusions suggest that the use of NLP methods can effectively assess body image perception disturbances in head and neck cancer patients, offering an empathic way to better understand and support patients' emotional and psychological well-being during their cancer journey. |
| Kharko et al (2024) [42] | To explore the effectiveness of OpenAI’s ChatGPT 3.5 and GPT 4.0 in generating patient-facing clinical notes from fictional general practice reports. Specifically, the study aimed to evaluate how these generative AI models influenced readability, sentiment, empathy, and medical fidelity in clinical documentation. With increasing patient access to online clinical records, the study aimed to determine if AI-generated notes could meet the needs of patients while maintaining accuracy and empathy. | Cross-sectional Study | Diagnostic, Tailored Patient Education, Tailored Provider Education, Patient Emotional Support, Caregiver Support, Care Plan Optimization, Palliative and End-of-Life Care | Conclusion suggests both ChatGPT 3.5 and GPT 4.0 generated longer notes compared to clinician-written reports, with ChatGPT 3.5 using more second-person pronouns, potentially creating a more personalized feel. While the AI models improved sentiment and empathy, making the notes more emotionally engaging, they also required higher reading proficiency. Additionally, there were concerns about medical fidelity, as some AI-generated notes omitted key details, potentially affecting their accuracy. GPT 4.0 outperformed 3.5 in medical fidelity, though both versions still showed limitations in the precision of medical information. |
| Klotz et al (2025) [57] | To assess the quality and effectiveness of ChatGPT responses compared to responses from experienced pancreatic surgeons when answering common patient queries about pancreatic cancer surgery. Specifically, the study aimed to evaluate the accuracy of the content, the clarity of the responses for non-specialist audiences, and the level of empathetic resonance conveyed by both AI and human responses. This investigation explores the potential role of AI in enhancing patient-clinician communication, particularly in the sensitive context of cancer care. | Comparative Cross-sectional Observational Study | Patient Emotional Support, Caregiver Support, Tailored Patient Education, Tailored Provider Education, Care Plan Optimization | Findings suggest that the responses from ChatGPT and the surgeons were comparable in several important areas. Both the AI and the surgeons were highly rated in terms of content reliability and clarity, with ChatGPT’s responses also receiving positive feedback for being easily understandable by non-specialists. In terms of empathy, ChatGPT’s responses were similarly evaluated as being empathetic, with patients rating the AI’s emotional resonance almost as high as that of the surgeons.  Interestingly, Surgeon 1’s responses were rated the highest, followed closely by those of ChatGPT, highlighting the potential for AI to replicate the empathetic communication traditionally provided by human clinicians. This suggests that AI has the capacity to enhance patient-provider communication by delivering not only accurate information but also an empathetic response that resonates with patients’ emotional needs. |
| Alowais et al (2023) [43] | To assess the role and potential of AI in revolutionizing clinical practice, including personalized healthcare, diagnosis, treatment, patient care, and emotional support, with a focus on the empathetic inclusion of AI in improving patient-provider relationships in the context of cancer care. | Narrative Review | Diagnostic, Patient Emotional Support, Caregiver Support, Tailed Patient Education, Tailored Provider Education, Care Plan Optimization, Palliative and End-of-Life Care | The review concludes that AI can significantly enhance clinical practice, particularly in cancer care, by not only improving diagnostic accuracy and treatment personalization but also supporting patient emotional well-being through empathic AI systems. These systems can foster stronger patient-provider relationships, reduce emotional distress in patients, and improve healthcare accessibility, thus emphasizing the importance of integrating empathy into AI technologies for a more holistic, human-centered care approach. |
| Adikari et al (2022) [53] | This study aims to develop and validate an empathic conversational agent framework to support patient mental health and wellbeing in patient-centered healthcare settings. | Pilot Study | Diagnostic, Patient Emotional Support, Caregiver Support | This study presented an empathic conversational agent framework that leveraged NLP techniques and AI algorithms to detect and analyze patient emotions, emotion transitions, group emotions, and behavioral metrics, demonstrating its effectiveness through empirical evaluations on benchmark datasets and clinical validation in online cancer support groups. While the framework proved valuable as an adjunct to healthcare practitioners, future improvements will focus on enhancing emotion extraction and contextual understanding using advanced NLP transformer-based architectures, further reducing the need for clinician intervention. |
| Adikari et al (2020) [54] | This study highlights the opportunity of using AI in Online Cancer Support Groups to address psychological morbidity, with supporting empirical evidence from prostate cancer (PCa) patients. | Retrospective Cohort Study | Patient Emotional Support  Care Plan Optimization | This research examined the impact of OCSGs on psychological distress among prostate cancer (PCa) patients using PRIME, an AI framework validated on a large dataset of 277,805 conversations involving 18,496 patients across ten international OCSGs. Findings revealed that pre-treatment participation improved emotional states, while long-term engagement enhanced overall emotional well-being. These results underscore the validity and healthcare value of AI in OCSGs as an effective tool for early psychological intervention alongside formal treatment processes. |
| Mårell-Olsson et al (2021) [44] | This study aims to examine the non-medical needs of children with long-term illnesses and explore how socially intelligent agents can enhance their interaction, participation, and quality of life. | Qualitative Semi-Structured Interview | Tailored Patient Education,  Patient Emotional Support | Conclusions suggest that the use of AI-based SIAs to enhance the quality of life for children with long-term illnesses can help to address their non-medical needs. It highlighted how AI tools can reduce feelings of isolation, strengthen participation in school activities, and improve interaction with teachers, classmates, and friends. By providing consistent explanations of treatments and encouraging healthy coping techniques, SIAs offer digital support that complements human warmth and connection, fostering inclusion and improving overall well-being for hospitalized children. |
| Chow and Li (2024) [45] | To critically examine the ethical considerations surrounding the use of LLMs, such as GPT-3 and GPT-4, in developing oncology chatbots. The study highlights the significance of human-centered AI, which prioritizes ethical principles, empathy, and a user-centric approach to address patient and family concerns in the oncology field. The review identifies key ethical challenges, particularly focusing on bias in AI outputs resulting from skewed training datasets and suggests strategies for mitigating these biases to ensure equitable healthcare delivery. | Narrative Review | Patient Emotional Support, Caregiver Support, Tailored Patient Education, Tailored Provider Education, Care Plan Optimization | Findings argue that while LLMs, like GPT-3 and GPT-4, hold significant potential for use in oncology chatbots, their development must be handled with ethical care to ensure they serve diverse patient populations equitably. The review emphasizes that bias in training data, such as an overrepresentation of Western perspectives, can result in outputs that fail to adequately serve marginalized or underrepresented groups. Therefore, the development of human-centered AI in oncology chatbots must prioritize ethical principles and empathy, incorporating safeguards to mitigate biases and ensure that these AI systems offer empathetic, precise, and culturally appropriate responses. The study advocates for inclusive AI design that aligns with human-centric values to provide better, more equitable healthcare outcomes. |
| Ranasinghe et al (2018) [46] | This study applies the PRIME-2 framework to analyze patient-reported outcomes from online cancer support group discussions, comparing robot-assisted laparoscopic prostatectomy (RARP) and open radical prostatectomy (ORP). The framework uses machine learning to identify and assess emotional, functional, and decision-making outcomes, offering empathic AI insights into patient sentiments and quality of life over time. | Retrospective Cohort Study | Patient Emotional Support, Tailed Patient Education, Tailored Provider Education, Care Plan Optimization | Conclusions suggest that RARP and ORP patients had similar side effects over 12 months, but emotions differed, with RARP patients reporting more positive, consistent emotions, while ORP patients expressed more negative emotions, particularly during surgery and follow-up periods. The PRIME-2 framework highlights the importance of empathic AI in recognizing and addressing emotional challenges, emphasizing its role in improving patient care and supporting emotional well-being throughout treatment. |
| Spiegel et al (2024) [47] | This study aims to assess the acceptability and safety of an AI-driven virtual reality therapy platform (XAIA) for individuals with mild-to-moderate anxiety or depression. | Pilot Study | Tailored Patient Education, Patient Emotional Support, Care Plan Optimization | Conclusions suggest that AI-driven virtual reality therapy is acceptable and safe, with participants engaging meaningfully with the system, indicating potential for digitally mediated emotional support, although human interaction remains preferred by some users. |
| Mao et al (2024) [48] | This study aims to evaluate the effects of a virtual reality–based mindfulness training program on anxiety, depression, and cancer-related fatigue in ovarian cancer patients undergoing chemotherapy. | Prospective Cohort Study | Tailored Patient Education, Patient Emotional Support | This study showed that ovarian cancer patients undergoing chemotherapy have a high acceptance of virtual reality-based mindfulness training mode. The application of this mode can reduce the psychological problems of anxiety, depression, and cancer-related fatigue in ovarian cancer patients during chemotherapy and is worth promoting and using. Given the decreasing price of virtual reality kits and easy installation of the model, the application of the Mindfulness Training Virtual Reality model could be extended from clinical psychological intervention to community-based or home-based psychological intervention under a reduced cost without the participation of professional medical staff. |
| Hamza-Cherif et al (2024) [49] | This study aims to compare machine learning and deep learning models for classifying emotional sentiment in textual data from patients with serious illnesses. | Comparative Study | Tailored Provider Education, Patient Emotional Support | The results highlight the superior performance of the long short-term memory (LSTM) and bidirectional encoder representations from transformers (BERT) deep learning models compared to the reference models. Even though the EmoHD database explored in this study is interesting in terms of application in cognitive psychology to help identify the mental state of patients suffering from serious illnesses and to promote a healing process by addressing negative thoughts of patients thanks to a dedicated diagnosis providing an automatic system based on robust learning models for sentiment analysis, EmoHD presents many limitations. |
| Bandaragoda et al (2018) [55] | This study aims to evaluate the PRIME framework, which uses machine learning and deep learning to analyze self-reported emotional and quality-of-life data from prostate cancer patients in online support groups. The framework identifies emotional challenges, offering empathic insights to tailor interventions and support emotional needs related to side effects and QoL. | Retrospective Cohort Study | Patient Emotional Support, Caregiver Support, Tailed Patient Education | The PRIME framework effectively identifies emotional and QoL challenges in prostate cancer patients, particularly younger patients and their partners. It demonstrates how empathic AI can provide personalized emotional support and detect specific psychosocial needs, improving care and identifying patients who require additional support. |
| Leung et al (2022) [50] | The purpose of this pilot study is to evaluate the development and effectiveness of an empathic artificial intelligence-based co-facilitator (AICF) integrated with a natural language processing (NLP) recommender system, designed to identify psychosocial challenges and emotional distress in cancer patients within online support groups. The AICF aims to provide empathic, real-time, tailored emotional support by suggesting relevant self-care resources to patients based on their expressed emotional and psychosocial needs. | Prospective Cohort Study; Pilot | Patient Emotional Support, Caregiver Support, Tailed Patient Education, Care Plan Optimization, Palliative and End-of-Life Care | Conclusion demonstrates that the empathic AI-based co-facilitator (AICF) can effectively identify emotional and psychosocial challenges in cancer patients participating in online support groups. With high precision, recall, and F1 scores, the system was successful in recommending personalized, empathetic support resources. By enhancing the emotional well-being of patients through tailored resource suggestions, the AICF showed significant promise in providing continuous empathic support beyond therapy sessions. The findings highlight the potential for empathic AI to augment cancer care by offering real-time emotional and psychosocial support that aligns with patients’ individual needs. |
| O’Gara et al (2022) [51] | The SafeSpace study sought to codesign and test a virtual reality (VR) psychological support intervention that integrates relaxation and compassionate mind training to determine its feasibility and acceptability in an oncology setting. The effect of the VR intervention on mental and physical health and quality of life was evaluated using a number of scales and questionaries as well as qualitative interviews. | Experience-based codesign (EBCD) and mixed methods | Diagnostic, Patient Emotional Support, Caregiver Support, Tailed Patient Education, Tailored Provider Education, Care Plan Optimization, Palliative and End-of-Life Care | Findings suggest that people affected by cancer (PABC) accepted the psychological VR intervention as a safe and feasible way to address their unique, unmet psychological needs. Stress reduction effects were noted in the oncology setting, and further exploration of a home-based, self-administered version is suggested. A larger study is also proposed to substantiate the findings. |
| Niki et al (2019) [56] | To verify whether using simulated virtual reality (VR) travel improves symptoms in terminal cancer patients. The VR trips featured memorable scenes or places the participant always wanted to go but never had. Physiological symptoms as well as emotional reactions were evaluated. | Prospective, multicenter, single-arm study | Patient Emotional Support, Palliative and End-of-Life Care | The study found that among those who went to 'memorable places' (75% of the cohort), significant improvements were made in terms of alleviating pain, fatigue, depression, anxiety, and emotional well-being, with depression showing the greatest effect size. The VR travel intervention also increased the participants' sense of fun and happiness. The remaining 25% of the cohort who went 'somewhere they always wanted to go' did not experience siginificant improvements, suggesting episodic memories involving the medial temporal lobe (MTL) played a role. |

**a.** Abbreviations: AI, artificial intelligence; NLP: Natural Language Processing; LLM: Large Language Model; QoL: Quality of Life; RARP: robot-assisted laparoscopic prostatectomy; ORP: open radical prostatectomy; OSGs: Online Support Groups
